# The uncorrected refractive error challenge

**Published:** 2014

**Authors:** Kovin Naidoo, Pirindha Govender, Brien Holden

**Kovin Naidoo, Pirindha Govender** and **Brien Holden**

Refractive error affects people of all ages, socio-economic status and ethnic groups. The most recent statistics estimate that, worldwide, 32.4 million people are blind and 191 million people have vision impairment.^1^ Vision impairment has been defined based on distance visual acuity only, and uncorrected distance refractive error (mainly myopia) is the single biggest cause of worldwide vision impairment. However, when we also consider near visual impairment, it is clear that even more people are affected. From research it was estimated that the number of people with vision impairment due to uncorrected distance refractive error was 107.8 million,^1^ and the number of people affected by uncorrected near refractive error was 517 million,^2^ giving a total of 624.8 million people.

Vision impairment affects the ability to function optimally, socialise and engage in activities of daily living and emotional well-being. In children, vision impairment is known to affect school learning, outdoor activity and the individual's social life or integration.^3^ Uncorrected distance refractive error leading to vision impairment can reduce quality of life^4^ and decrease participation in daily activities that are vision-related.^5^ Uncorrected near vision also reduces an individual's educational and employment opportunities.^6^ Uncorrected refractive error has broader implications for communities, countries and the global community. The potential lost productivity as a result of uncorrected distance refractive error is US $268.8 billion per year. The cost to train refractionists and maintain refractive services to deal with uncorrected refractive error (including presbyopia) is US $28 billion.^7^

Despite being one of the more easily corrected conditions resulting in vision impairment, uncorrected refractive error still remains a significant cause of vision impairment globally. A number of factors contribute to this situation: a lack of trained eye health workers to address the current refractive challenges, poor integration of refractive services into existing eye health services and a limited number of good quality training programmes.

## Key strategies in addressing the problem

### Human resource development

The World Health Organization's Global Action Plan for 2014 to 2019 has identified human resources for refractive error as a priority in reducing avoidable blindness globally.^7^,^8^ Current challenges include the uneven distribution of refraction training institutions and a lack of standardisation, which makes it difficult to maintain the quality of services. In some countries, competing eye health priorities also mean that providers sometimes neglect refractive error services.

### Service delivery

In many low- and middle-income countries, it is necessary to provide refractive services at all levels of the health care system, especially at primary level, where services are provided in the community. Successful services have an integrated team approach, with a clear referral pathway and a defined scope of service at each level. For example: screening/case finding at community level, presbyopia or basic refraction services at primary or community health centre level, specialised services at secondary or district level, and pre- and post-operative refraction services at tertiary or regional level.

### Social enterprise

Social enterprise (SE) solutions provide refractive error services while at the same time alleviating poverty and providing employment opportunities. SE initiatives are meant to complement existing eye care delivery systems, and can take many forms. A vision centre model charges those who can pay and uses this income to subsidise services for the poorest of the poor and is usually run by NGOs or in partnership with the public sector. A social franchise model allows entrepreneurs to be supported to make affordable frame and lens packages available in under-served areas.

**Figure:**
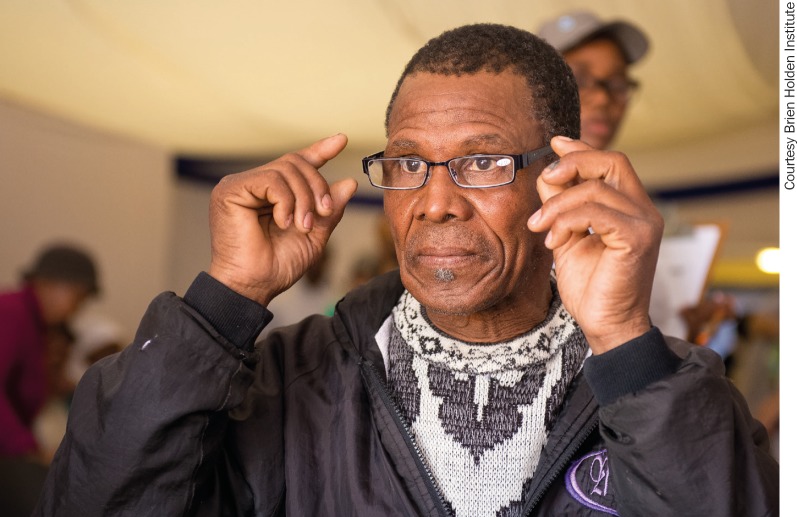
Uncorrected distance refractive error is the biggest single cause of vision impairment worldwide. SOUTH AFRICA

### Infrastructure and supplies

Delivering comprehensive, accessible eye care to communities means that the necessary equipment and space needs to be allocated for services to be delivered and an affordable spectacle supply chain should be in place. In some cases, refractive services are provided, but an inadequate supply of spectacles makes these services irrelevant as people still have to live with uncorrected refractive error.

**“Successful services have an integrated”**

It is evident that there is still much that should be done to alleviate the problem. By developing evidence through research initiatives (e.g. determining the regional and country-specific prevalence of uncorrected refractive error or mapping human resources), country-specific solutions can alleviate the problem in a comprehensive and coherent manner. Research data on the impact correcting refractive error has on people's education and socioeconomic status will provide the information needed for successful advocacy efforts.

**Figure:**
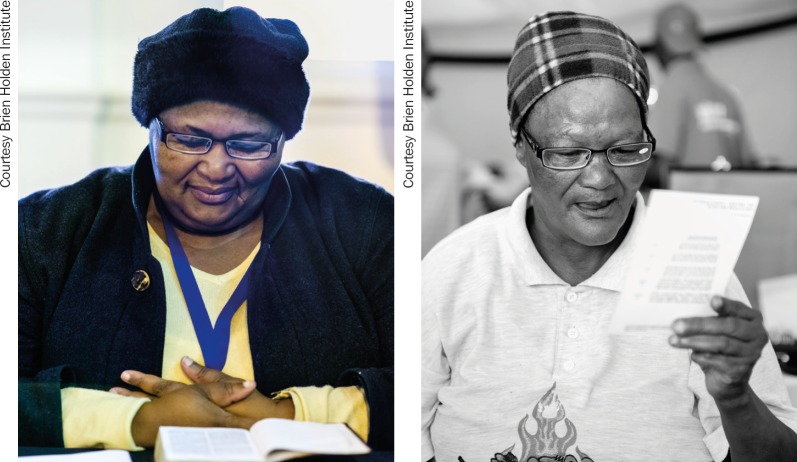
An estimated 517 million people worldwide are affected by uncorrected near refractive error, which reduces their employment opportunities. SOUTH AFRICA
